# Diversity of Avian leukosis virus subgroup J in local chickens, Jiangxi, China

**DOI:** 10.1038/s41598-021-84189-7

**Published:** 2021-02-26

**Authors:** Haiqin Li, Meifang Tan, Fanfan Zhang, Huayuan Ji, Yanbing Zeng, Qun Yang, Jia Tan, Jiangnan Huang, Qi Su, Yu Huang, Zhaofeng Kang

**Affiliations:** 1 Institute of Animal Husbandry and Veterinary Medicine, Jiangxi Academy of Agricultural Sciences, Nanchang, 330200 Jiangxi China; 2 Institute of Animal Husbandry and Veterinary Medicine, Fujian Academy of Agricultural Sciences, Fuzhou, 350013 Fujian China; 3 College of Veterinary Medicine, Shandong Agricultural University, Tai’an, 271000 Shandong China

**Keywords:** Pathogens, Virology

## Abstract

Avian leukosis caused by avian leukosis virus (ALV) is one of the most severe diseases endangering the poultry industry. When the eradication measures performed in commercial broilers and layers have achieved excellent results, ALV in some local chickens has gradually attracted attention. Since late 2018, following the re-outbreak of ALV-J in white feather broilers in China, AL-like symptoms also suddenly broke out in some local flocks, leading to great economic losses. In this study, a systematic epidemiological survey was carried out in eight local chicken flocks in Jiangxi Province, China, and 71 strains were finally isolated from 560 samples, with the *env* sequences of them being successfully sequenced. All of those new isolates belong to subgroup J but they have different molecular features and were very different from the strains that emerged in white feature broilers recently, with some strains being highly consistent with those previously isolated from commercial broilers, layers and other flocks or even isolated from USA and Russian, suggesting these local chickens have been acted as reservoirs to accumulate various ALV-J strains for a long time. More seriously, phylogenetic analysis shows that there were also many novel strains emerging and in a separate evolutionary branch, indicating several new mutated ALVs are being bred in local chickens. Besides, ALV-J strains isolated in this study can be further divided into ten groups, while there were more or fewer groups in different chickens, revealing that ALV may cross propagate in those flocks. The above analyses explain the complex background and future evolution trend of ALV-J in Chinese local chickens, providing theoretical support for the establishment of corresponding prevention and control measures.

## Introduction

Avian leukosis virus (ALV) belongs to the genus *alpharetrovirus* of the family *retroviridae* and causes avian leukosis as the first known virus-related tumor diseases, leading to great economic losses^1,2^. Up to now, 11 different subgroups of ALV (designated A to K) have been determined based on host range, envelope properties as well as cross-reactivity, and those in subgroups A, B, C, D, E, J and K are capable of infecting chickens^3–6^. Among them, the strains of subgroup E are endogenous and non-pathogenic^7^, while that of subgroup J (ALV-J) is the most prevalent and causes the myelocytoma, hemangioma and multiple other malignant tumors^1,8^. In China, ALV-J was first detected in 1999 and then spread rapidly throughout the country, which used to be a major disease endangering China’s poultry industry and posed a huge threat to the supply of eggs and chicken products for a long time^9,10^.

To reverse this situation, a nationwide eradication program for controlling ALV infection had been launched by China’s government since 2008 and finally achieved great success, especially in commercial broiler and layer chickens^11^. Currently, the morbidity of AL was significantly reduced and the pandemic was universally limited, which is particularly obvious for ALV-J. And, after 2013, there are few reports about the outbreak of AL in China. After that, more attention has been paid to the eradication of ALV in Chinese local chickens, where have more complicated infection status, and there are often many different subgroups of ALV mixed, raising a lot of recombinant strains^12–18^. Fortunately, the pathogenicity of the epidemic strains in the local chickens may be relatively weak or these chickens have some natural resistance to ALV, which makes them not have a large number of deaths and serious AL outbreaks in the past^19–21^.

However, this does not mean that ALV is no longer a problem. Since 2018, there has been another outbreak of myelocytomatosis of unknown origin in white feather broilers caused by several mutational ALV-J isolates in China, which have a lot of newly emerged genomic features that may be related to increased pathogenicity and altered histotropism^22,23^. At the same time, similar symptoms of AL have also been found in many local chicken farms in China, but the corresponding pathogen has not been identified. Considering that the previous ALV strains circulating in Chinese local chickens are mainly subgroups A, B, J and K with relatively lower pathogenicity^24,25^, it is possible that the epidemic ALV strains in Chinese local chickens have changed, which deserves further analysis.

For verifying the molecular characteristics of ALV which recently broke out in local chicken farms in China and to clarify its potential transmission route, a systematic epidemiological investigation was carried out in eight local chicken farms in Jiangxi Province, China, to lay a foundation for the development of the corresponding control measures.

## Results

### Clinical symptoms, post-mortem and histopathology findings

**Figure 1:**
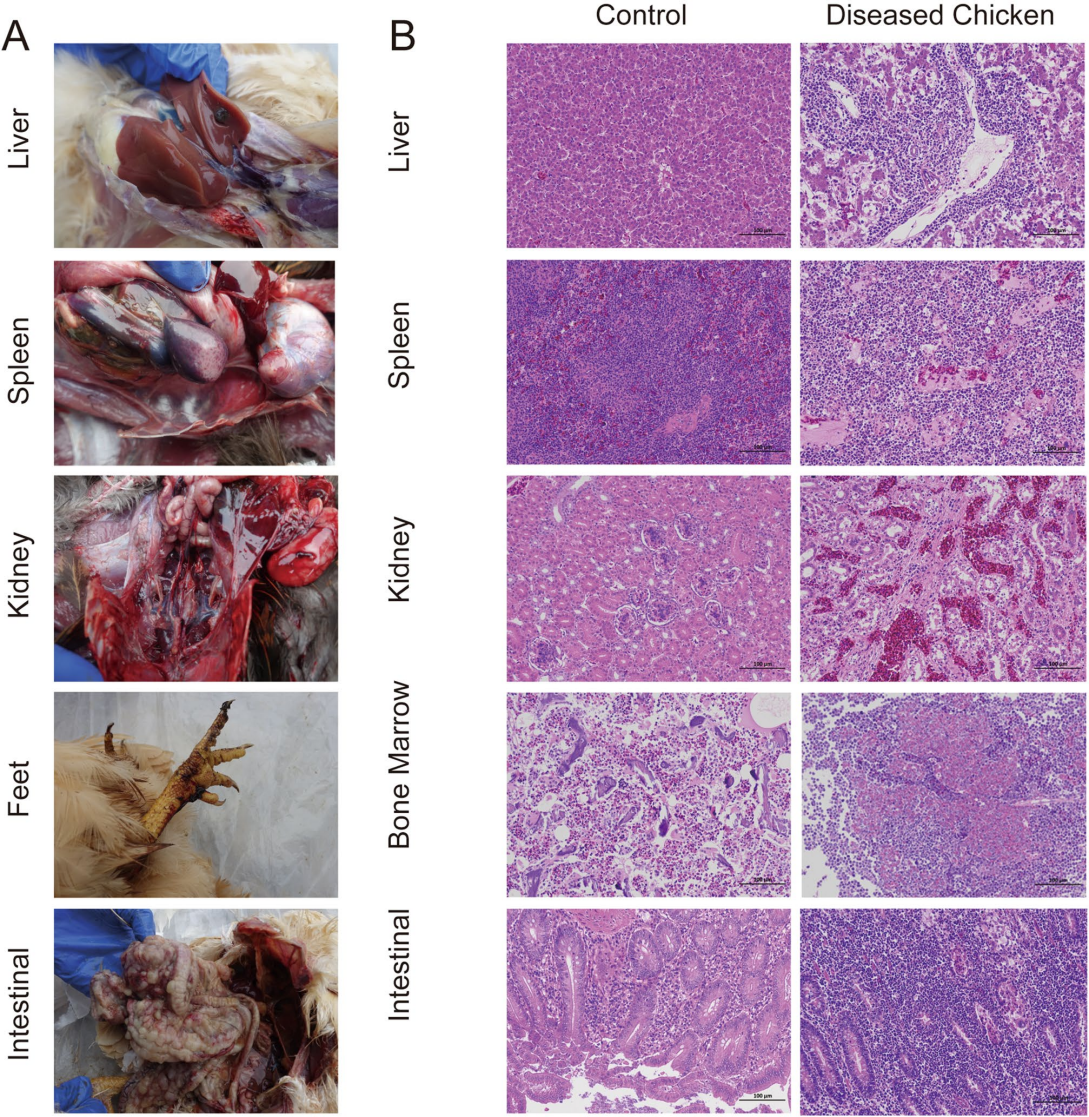
Anatomical and histological examination of sampled chicken. (**A**) Sampling chicken's liver, spleen, kidney, feet and intestinal; (**B**) pathological sections of diseased chicken and control (H&E stain, 400 ×).

The main clinical signs and post-mortem lesions presented by the sampled chickens were enlarged liver with obvious hemorrhagic sac, splenomegaly with white tumor nodule, renal enlargement, foot hemorrhage, and mesentery tumor (Fig. 1A). As shown in Fig. 1B, the liver has obvious inflammatory cell infiltration and tumor cells. In addition, no obvious white marrow structure was found in the spleen with loosely arranged cells. A large number of myeloid tumor cells and eosinophils can be found in the blood vessels, while many lymphoid cells appeared in the interstitium. Also, several myeloid cells dyed in red lymphoid cell clusters. No trabecular bone structure was found in the bone marrow tissue where suspicious tumor cells were scattered. A large number of diffuse lymphoid cells can be seen in the local lamina propria of the intestine. Many myeloid tumor cells and a few intestinal epithelial cells are necrotic. Uninfected healthy chicken tissue was used as a negative control for histopathological diagnosis.

### Isolation and Identification of ALV

**Figure 2:**
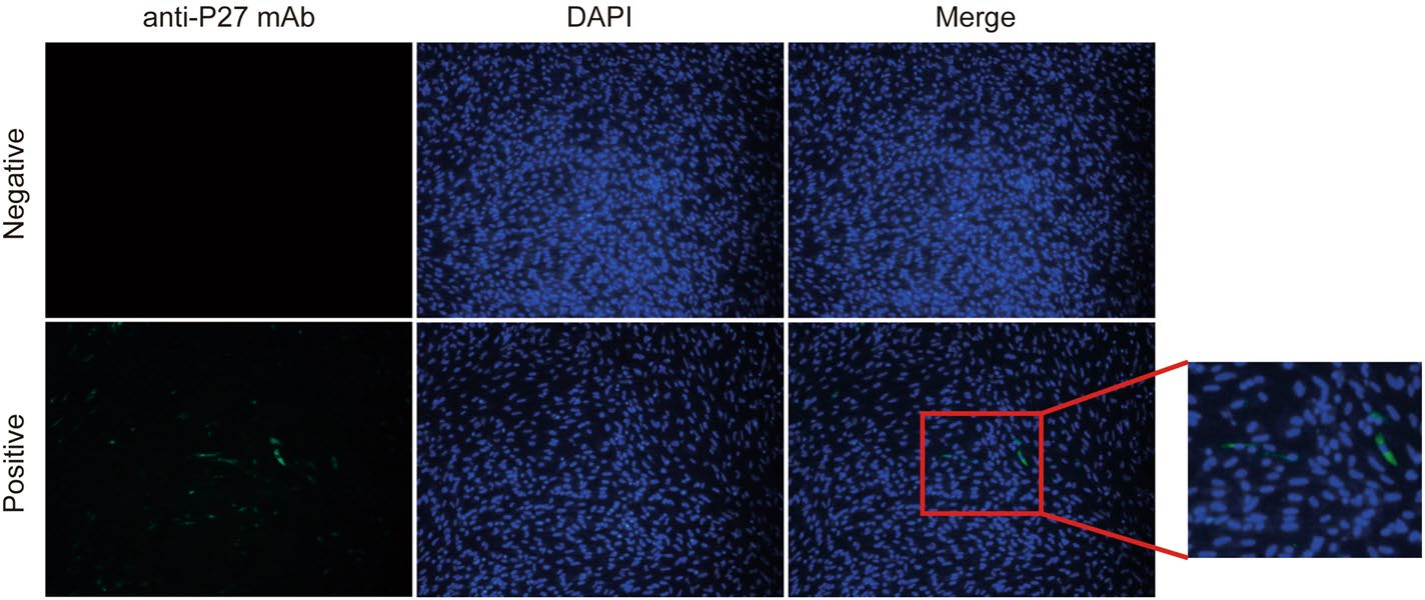
IFA recognizes ALV infection in incubated cells. At 7 days after incubation, the cells were fixed and an IFA was performed with a mAb against P27. DAPI were used to stain the nucleus.

Seventy-one ALV strains were finally isolated from the above analysis using DF-1 cells combined with Anti-P27 ELISA assays and IFA. Among them, 58 strains were directly determined through ELISA assays, and DF-1 cells infected with these strains showed a positive result by measuring the ALV P27 antigen (the ELISA S/P values (Samples OD value-negative control value/(positive control value-negative control value)) of the positive samples ranged from 0.29 to 1.27, respectively, while positive critical value was 0.2). During the above analysis, this study also found that the results of fifteen samples are between 0.17 and 0.2, and they may also be positive. For further identifying the ALV in corresponding incubated DF-1 cells, IFA using mAb against P27 was also employed in this study. Finally, the specific green fluorescence was observed in the cells infected with ALV, which turns out that 13 of them were ALV positive, although only a few cells were infected (Fig. 2). In a nutshell, a total of 71 strains were isolated from 560 samples with a positive rate at 12.68%, while there is a big difference in the positive rate among different farms. The highest positive rate can be found in the farm of Anyi Tile-like Gray Chicken as 29%, compared with only 1.67% in that of Chongren Chicken, while others have a positive rate from 2.00 to 19.00% (Table 1).

**Table 1:** ALV-Positive rate in the farms investigated in this study.

Breed name	Samples collected	Positive samples	Positive rate (%)
Ningdu yellow chicken	70	3	4.29
Taihe silky fowl	50	9	18.00
Anyi tile-like grey chicken	100	29	29.00
Dongxiang blue-shell chicken	100	19	19.00
Chongren chicken	60	1	1.67
Guangfeng baier yellow chicken	60	7	11.67
Xiushui black-bone chicken	70	2	2.86
Yugan black chicken	50	1	2.00
Total	560	71	12.68

### Subgroup identification and phylogenetic analysis

After genomic DNA extraction, samples tested positive by ELISA or IFA were all positive by ALV-J specific primers, while no positive appeared using ALV A-E and K subgroup-specific primers, which primarily indicated that all these 71 isolates belonged to subgroup J. After that, the *env* gene of them was successfully sequenced in this study and then named as “province-year-location-number”. Corresponding sequences have been submitted into Genbank, and the accession number for them can be found in Table 2.

**Table 2:** ALV-J isolated in this study and one to one corresponding the most similar reference strain published in Genbank.

ALV-J isolated in this study	Most similar known strain	Similarity (%)	Time	Location	Source
JX19DX06-MN262574	ZB110604-3-KC841154	100	2011	Shandong	Chicken
JX19AY19-MN262558	GD13HY-KU500031	100	2013	Guangdong	Yellow broiler chicken
JX19AY17-MN262556	JS16JH3-MG700535	100	2016	Shandong	Gallus gallus Black-bone
JX19AY11-MN262550	LC110515-3-KC841152	100	2011	Shandong	Chicken
**JX19AY09-MN262548**	JS09GY5-GU982309	100	2009	Jiangsu	Commercial layer chicken
**JX19AY06-MN262545**	GX-J-6-JQ246095	100	2010	Guangxi	Chicken
**JX19AY05-MN262544**	GX-J-7-JQ246096	99.78	2010	Guangxi	Chicken
JX19XS01-MN262595	GDQY1201-JX423792	99.68	2012	Guangdong	Yellow meat-type chicken
JX19AY13-MN262552	ZB110604-6-KC841157	99.67	2011	Shandong	Chicken
JX19DX07-MN262575	SDAU1706-KY980662	99.36	2017	Shandong	Hy-line chicken
**JX19AY21-MN262560**	2012004-C5-KC453974	99.36	2012	Guangxi	Chicken
JX19AY08-MN262547	JS09GY5-GU982309	99.35	2009	Jiangsu	Commercial layer chicken
**JX19AY24-MN262563**	CLB908U-JQ935966	99.24	2009	**Russia**	Gallus gallus
JX19AY16-MN262555	GX-J-8-JQ246097	99.04	2010	Guangxi	Chicken
JX19DX16-MN262584	JS16JH10-MG700542	98.83	2016	Shandong	Gallus gallus Black-bone
JX19AY15-MN262554	GX-J-8-JQ246097	98.61	2010	Guangxi	chicken
JX19AY29-MN262568	GD06SL4-EF103131	98.60	2006	Shandong	Chinese 'yellow' chickens
**JX19DX15-MN262583**	JS16JH10-MG700542	98.51	2016	Shandong	Gallus gallus Black-bone
JX19DX02-MN262570	JS16JH2-MG700534	98.27	2016	Shandong	Gallus gallus Black-bone
JX19AY23-MN262562	UD5-AF307952	98.07	2001	**USA**	Chicken
JX19GF05-MN262592	sdau1002-JN389518	97.86	2010	Shandong	Layer chicken
JX19ND02-MN262538	GX13DF52-KU848768	97.83	2013	Guangxi	Chicken
JX19AY10-MN262549	LC110515-3-KC841152	97.74	2011	Shandong	Chicken
**JX19DX19-MN262587**	sdau1002-JN389518	97.64	2010	Shandong	Layer chicken
JX19DX01-MN262569	GX14HG01-KU997685	97.63	2014	Guangxi	Chicken
**JX19TH08-MN262535**	GX13DF52-KU848768	97.18	2013	Guangxi	Chicken
JX19TH03-MN262530	GX13DF52-KU848768	97.18	2013	Guangxi	Chicken
JX19DX12-MN262580	SDAU1706-KY980662	97.11	2017	Shandong	Hy-line chicken
**JX19YG01-MN262598**	GX14LT07- KX034517	97.07	2014	Guangxi	Chicken
**JX19ND03-MN262539**	WA1112-KJ631315	96.96	2012	Guangdong	Broiler breeder
JX19TH02-MN262529	WA1112-KJ631315	96.85	2012	Guangdong	Broiler breeder
JX19TH01-MN262528	WA1112-KJ631315	96.85	2012	Guangdong	Broiler breeder
JX19DX13-MN262581	SDAU1706-KY980662	96.79	2017	Shandong	Hy-line chicken
**JX19DX10-MN262578**	NHH-HM235668	96.77	2010	Jiangsu	Layer chicken
JX19TH05-MN262532	WA1112-KJ631315	96.74	2012	Guangdong	Broiler breeder
JX19TH04-MN262531	WA1112-KJ631315	96.53	2012	Guangdong	Broiler breeder
JX19AY14-MN262553	GD14J2-KU500032	96.45	2014	Guangdong	Yellow broiler chicken
JX19AY03-MN262542	PDRC-59831-KP284572	96.23	2007	**USA**	Gallus gallus
JX19DX05-MN262573	GX14YYD2-KU937324	96.16	2014	Guangxi	Chicken
JX19GF06-MN262593	M180-KX611834	96.13	2016	Guangdong	Chinese yellow chicken
JX19DX11-MN262579	NHH-HM235668	96.12	2010	Jiangsu	Layer chicken
JX19GF07-MN262594	M180-KX611834	95.91	2016	Guangdong	Chinese yellow chicken
JX19AY02-MN262541	GD0510A-EF103132	95.89	2016	Gongdong	Chinese 'yellow' chickens
JX19AY27-MN262566	XX2-09-HM775331	95.75	2010	Guangdong	Chicken
JX19AY26-MN262565	WN100401-HQ271447	95.39	2010	Anhui	Chicken
JX19AY22-MN262561	GX13ZS02-KY983561	95.22	2013	Guangxi	Gallus gallus
**JX19ND01-MN262537**	WA1112-KJ631315	95.11	2012	Guangdong	Broiler breeder
**JX19AY18-MN262557**	XX2-09-HM775331	94.91	2010	Guangdong	Chicken
JX19AY04-MN262543	GX13ZS12-KY983563	94.90	2013	Guangxi	Gallus gallus
JX19AY12-MN262551	ZB110604-6-KC841157	94.88	2011	Shandong	Chicken
JX19AY01-MN262540	GDQY1201-JX423792	94.85	2012	Guangdong	Yellow meat-type chicken
JX19AY20-MN262559	WN100401-HQ271447	94.50	2010	Anhui	Chicken
JX19AY28-MN262567	WSC112-KJ631322	94.45	2012	Guangdong	Broiler breeder
JX19TH07-MN262534	AF88-AF247390	94.30	2016	**USA**	Chicken
JX19TH09-MN262536	AF88-AF247390	94.04	2016	**USA**	Chicken
JX19AY07-MN262546	GX14HG04-KX058878	93.90	2014	Guangxi	Chicken
JX19TH06-MN262533	AF88-AF247390	93.40	2016	**USA**	Chicken
JX19DX17-MN262585	HuB09-1-HM600813	93.35	2009	Beijing	Chicken
JX19DX08-MN262576	GX14HG04-KX058878	93.27	2014	Guangxi	Chicken
JX19GF02-MN262589	GX14YL03- KT598470	92.95	2014	Guangxi	Chicken
JX19GF03-MN262590	GX15MM6-1-KU923584	92.84	2015	Guangxi	Gallus gallus
JX19DX14-MN262582	GX15MM6-1-KU923584	92.65	2015	Guangxi	Gallus gallus
JX19DX03-MN262571	SDAU1706-KY980662	92.60	2017	Shandong	Hy-line chicken
JX19DX04-MN262572	GX14YL03- KT598470	92.59	2014	Guangxi	Chicken
JX19DX18-MN262586	GX15MM6-1-KU923584	92.52	2015	Guangxi	Gallus gallus
JX19CR01-MN262597	SD110503-KF562374	92.37	2011	Shandong	Gallus gallus
JX19GF04-MN262591	GX15MM6-1-KU923584	91.97	2015	Guangxi	Gallus gallus
JX19GF01-MN262588	GX15MM6-1-KU923584	91.97	2015	Guangxi	Gallus gallus
JX19DX09-MN262577	SDAU1706-KY980662	91.96	2017	Shandong	Hy-line chicken
JX19AY25-MN262564	GDQY1201-JX423792	91.94	2012	Guangdong	Yellow meat-type chicken
JX19XS02-MN262596	GX14YL03- KT598470	91.72	2014	Guangxi	Chicken

The geographic location of the isolates in China is accurate to the provinces. The names of 13 strains that might replicate more slowly were bold.

For further identifying the subgroup of these 71 isolates, online BLAST program (https://blast.ncbi.nlm.nih.gov/Blast.cgi) was used to compare and analyze their similarity with all the published ALV strains, and the information of the reference strain with the highest similarity was recorded for analysis. As shown in Table 2, all of 71 strains shared the highest similarity with ALV-J reference strain, which confirmed again that they belong to the same subgroup. More importantly, six strains isolated in this study (JX19DX06, JX19AY19, JX19AY17, JX19AY11, JX19AY09, JX19AY06) have an incredible 100% similarity to the most corresponding similar strains isolated before, respectively. What's more, five of the above six strains were from the Anyi tile-like grey chicken, and they are 100% similar to the strains isolated at different times (2009, 2010, 2011, 2013, 2016), places (Guangdong province, Shandong province, Jiangsu province, and Guangxi province) and chicken species (yellow broiler chicken, Gallus gallus Black-bone and commercial layer chicken), indicating the wide distribution of these strains in space and time as well as the complexity of the source of ALV-J strains in the Anyi Tile-like Gray Chicken. Furthermore, the analysis also found that three strains isolated from Anyi Tile-like Gray Chicken have the highest similarity with several foreign epidemic strains, including JX19AY24 and CLB908U-JQ935966 (Russia, 2009, 99.24%); JX19AY23 and UD5-AF307952 (the USA, 2001, 98.07%); JX19AY03 and PDRC-59831-KP284572 (the USA, 2007, 96.23%), suggesting these relatively old strains still exist in China. The same phenomenon also appeared in the strains isolated from Taihe Silky Fowl, while three of them are the most similar to a strain isolated from the USA (AF88-AF247390, 2016, 93.40–94.30%). On the other hand, the time horizon of these most similar strains is very large from 2001 to 2017, which suggested that these strains may have continued to enter the chicken flocks we investigated and gradually accumulate to the current situation. Briefly, based on this analysis, it is found that the most similar reference strains of different strains isolated from the same farm in this study had great differences in time, location, and chicken species, which indicates that there are very complex epidemic situations and mixed infection in these farms.

**Figure 3:**
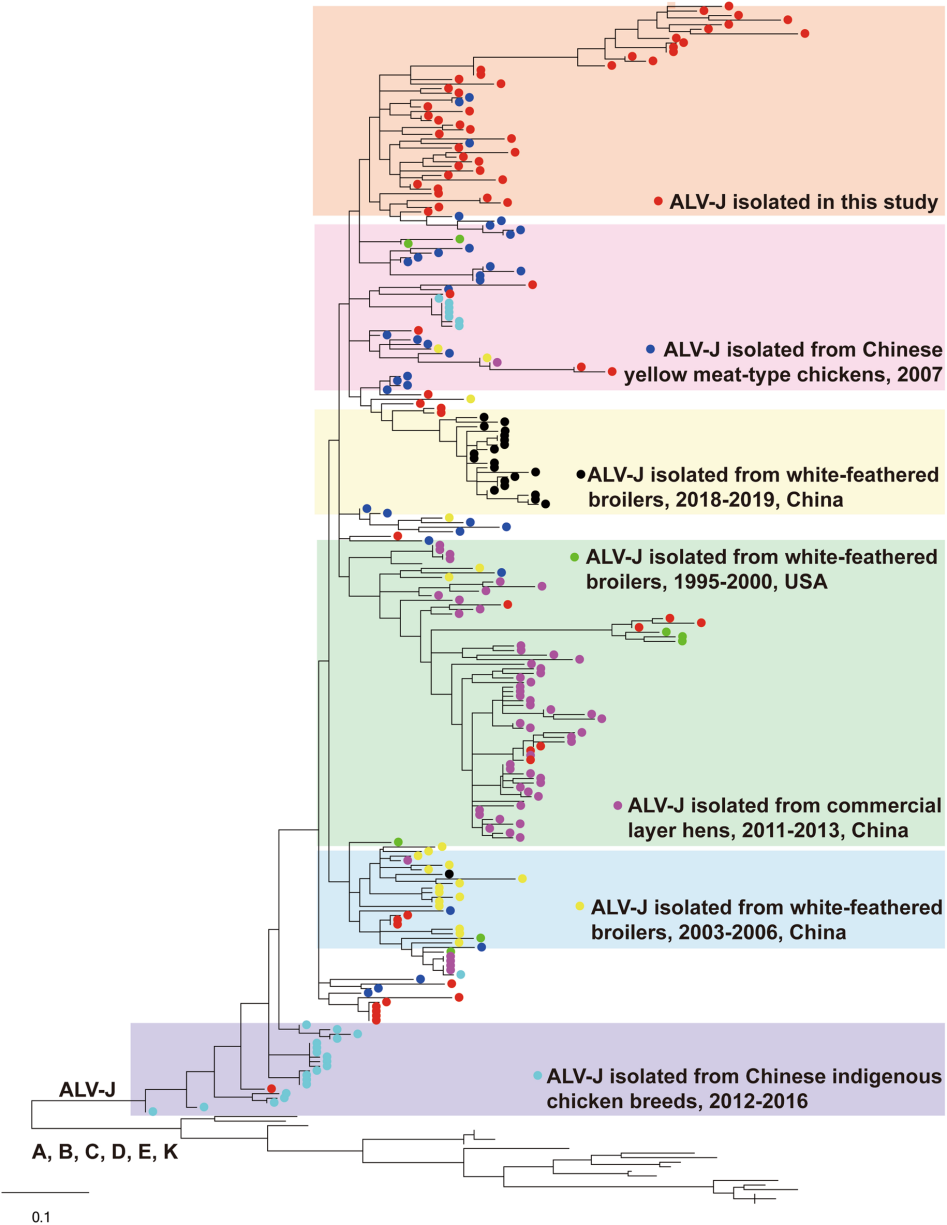
Phylogenetic tree based on the gp85 sequences of 71 ALV strains isolated in this study and 180 reference strains (162 ALV-J strains isolated from different flock and 18 ALV strains in subgroup A, B, C, D, E and J). The tree was constructed by the maximum likelihood method with 1000 bootstrap replicates using MEGA 5.0. The 71 ALV strains isolated in the study are shown with filled red circles, while other strains with different backgrounds are marked with different colored circles (specified in the figure).

To further clarify the relationship between isolates in this study and published reference strains, a total of 172 ALV-J strains as well as 18 isolates in subgroups A-E and K isolated over the past 30 years were collected from NCBI for analysis. Phylogenetic analysis based on the gp85 gene of new isolates and reference strains further confirmed that all these 71 strains were in the same branch with the reference ALV-J and had a relatively remote relationship with other subgroups (Fig. 3). More importantly, the analysis found that 43 new isolates converged in the same sub-branch on the top of the tree, while the other 28 strains were mixed with other strains with different backgrounds, which further explains the molecular diversity and source complexity of the virus strains this study isolated. Meanwhile, no strain is in the same branch as ALV-J which recently broke out in white-feathered broilers, indicating that the outbreak of ALV-J in the recent local chicken flocks may not be related to it. Besides, the genetic relationship of the strains isolated in this study is far from that of the strains isolated from Chinese indigenous chicken breeds in 2016, and the diversity of the strains isolated at that time was relatively low, indicating that the infection situation of ALV-J in Chinese local chickens in recent years has become increasingly complex.

On the other hand, it can also be observed that ALV-J isolated from Chinese indigenous chicken breeds in 2012–2016 is in a relatively primal position during genetic evolution, while the newly isolated ALV-J is relatively emerging with reference strains isolated from many types of chickens, and even 14 new isolates are in a further evolutionary fulcrum (the top of the evolutionary tree), suggesting that the infection status of ALV-J in the local chickens was constantly changing.

### Analysis of the diversity of 71 strains

**Figure 4:**
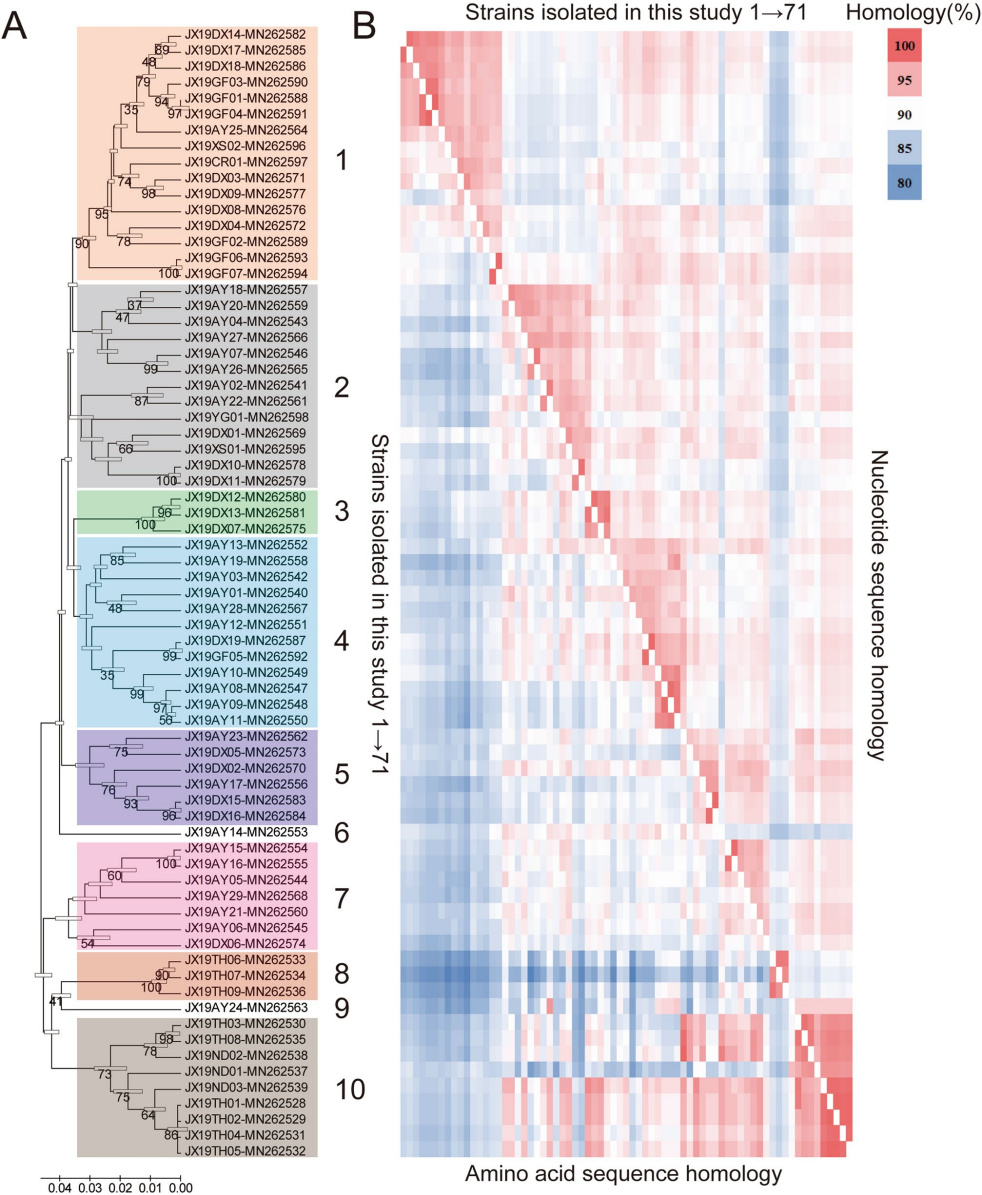
Sequences analysis of 71 ALV-J strains isolated in this study. (**A**) Phylogenetic analysis based on the env sequences of 71 ALV strains isolated in this study using the maximum likelihood method with 1000 bootstrap replications. The bar indicates genetic distance; strains in the same branch are labeled with the same color using Adobe Illustrator CS6; (**B**) similarity analysis heat map of 71 ALV strains isolated in this study; on both sides of the main figure is the virus strains, and in the middle is the similarity value (upper right: similarity of nucleotide sequences; lower left: similarity of amino acid sequences); different identity values are expressed in progressive colours ranging from 80 to 100%.

**Figure 5:**
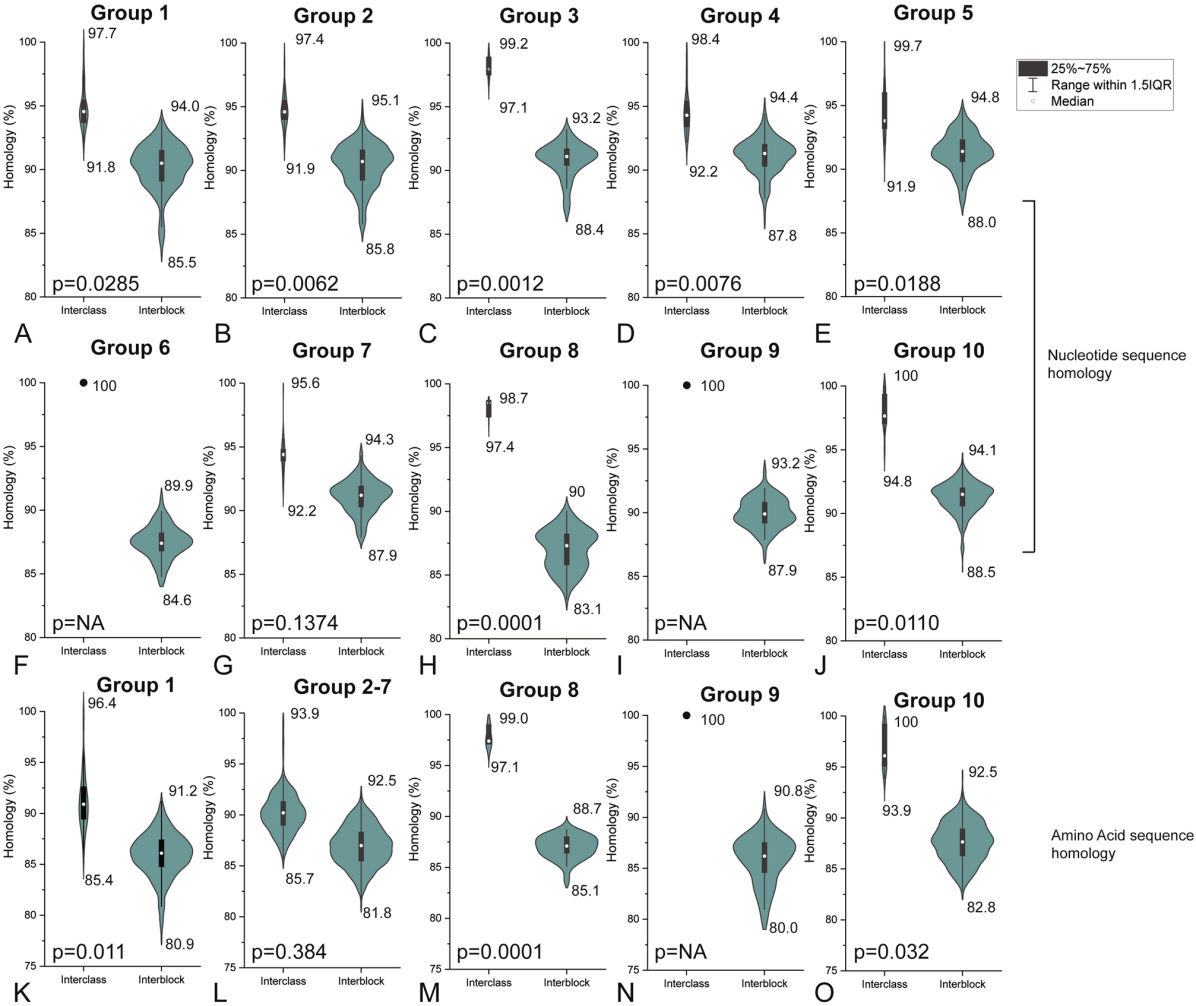
Comparison of interclass and interblock similarity of different groups of ALV-J strains isolated in this study. P value is calculated by t test. NA means not applicable. This image was drawn using GraphPad Prism 7.0.

To deeply understand the molecular characteristics of the 71 strains isolated in this study, another phylogenetic analysis based on the whole *env* gene was carried out to show the relationship between different new isolates. These 71 strains converge into ten sub-branches according to the phylogenetic analysis (Fig. 4A), indicating that some of them are more closely related to others during evolution. More importantly, the strains in the same sub-branch do not always come from the same farm, which means that the strains with the same origin may enter different farms in various ways. Furthermore, as shown in Fig. 4B, these strains in the same sub-branch share higher nucleotide sequence similarity, but the lower similarity with strains in other sub-branches. On the other hand, the similarity comparison based on the amino acid sequence also supports the above view, but the difference among groups 2–8 is not obvious. Detailed statistics about the interclass or interblock similarity can be found in Fig. 5. For example, the similarity of three new isolates (JX19TH06, JX19TH07 and JX19TH09, most similar to AF88 isolated in the USA) in group 8 were 97.4–98.7%, which was significantly higher than that between them and other strains (83.1–90%, P = 0.0001), showing that these strains are more closely related.

Furthermore, we also correlate the results in Table 1, Figs. 4 and 5, and found that the strains in groups 1–4 were mainly unrelated to ALV-J from other sources; the strains in group 5 are very close to those isolated from Chinese indigenous breeds; the strains in group 7 and 10 are very close to those isolated from Chinese yellow meat-type chickens; the strains in group 8 are very close to those isolated from the USA.

### Distribution of isolates in different sub-branches

According to the above analysis, the 71 strains we isolated can be divided into ten groups. As shown in Table 3, the farms in Anyi City (n = 7) and Dongxiang City (n = 6) have more groups, indicating a high diversity of ALV in them, which is consistent with our analysis above. Besides, other farms have fewer groups, just one or two. On the other hand, Group 1 and Group 2 are the most widely distributed, and both of them have been identified in five farms, respectively, while the distribution of other groups of viruses is slightly lower than that of group 1, appearing in one to three farms. Meanwhile, the above results also show that ALV-J currently circulating in Chinese local chickens were some newly emerged strains that have never been identified.

**Table 3:**
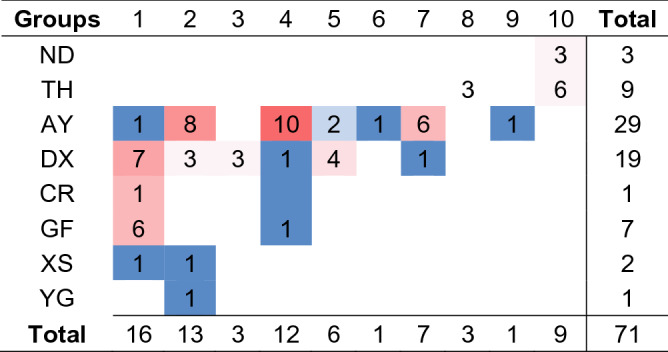
Distribution of different groups of ALV strains in different farms.

The same number is covered with the same color.

## Discussion

ALV is a naturally oncogenic pathogen that causes neoplastic diseases in poultry, such as lymphocytoma, myeloid leukosis, hemangioma, leading to great economic losses^1,2^. As a highly variable retrovirus, ALV can be classified as non-pathogenic endogenous strains (ALV-E) and exogenous strains that cause infections in a variety of birds. Among the latter, subgroups A, B and K mostly occur in indigenous chicken breeds with rare reports concerning subgroups C and D^4,14,26,27^, while ALV-J, being capable of inducing severe tumors and related symptoms in chicken, used to be the main cause of AL outbreak in commercial broilers and layers^28,29^. As a virus mainly spread vertically^30^, ALV can be controlled by strict provenance eradication measures, which has been carried out in many commercial flocks and achieved great achievements in a lot of countries^11^.

In China, Nationwide Eradication Program was also employed to restrain the epidemic of ALV in white feature boilers and commercial layers, and it has also achieved remarkable results since 2013, after which there have been very few AL outbreaks, further showing the effectiveness of provenance eradication measures. After that, more attention has been paid to local chicken breeds in China, and corresponding epidemiological analysis showed that there were more complicated infections in those flocks, namely, the mixed infection of ALV strains in different subgroups and the existence of recently identified ALV-K, even spawning a lot of recombinant strains^13–18^. Fortunately, years of observation showed that ALV did not cause a widespread and severe outbreak of AL in Chinese local chickens, and the ALV strains in many local chickens were very fixed and have obvious regional characteristics^31^, suggesting that these strains may coexist with corresponding chicken flocks for a long time or that these specific breeds have some resistance to previously existing ALV strains.

However, in the recent 2 years, there has been a big reversal of the above situation. ALV-J with mutated genome and increased pathogenicity has appeared in white-feathered broilers in many provinces of China since 2018, and these strains are also in relatively novel evolutionary branches, indicating that such emerged ALV-J from unknown sources has suddenly invaded the white broiler chicken, while the infections and epidemics arel continuing^22,23^. A little later, many Chinese local chickens began to show significant AL symptoms, accompanied by increased mortality and a significant drop in egg production, which leads us to think that there are also some new highly pathogenic ALVs in it. To this end, this study carried out a systematic epidemiological investigation on the local chickens in eight different cities in Jiangxi Province and analyzed the molecular characteristics of the current epidemic strains in detail.

First of all, this study noticed that the clinical symptoms of local chickens with suspected ALV infection were very similar to that of commercial layers and broilers, i.e. hepatosplenomegaly and scattered tumor nodules^8,22^, while histopathology analysis also showed that there was a lot of lymphocyte infiltration in various tissues and organs of diseased chickens, showing that different chicken breeds with ALV infection will show similar symptoms, and these fixed clinical features can further help us to diagnose ALV infection initially. After that, classical virus isolation using DF-1 cells and P27 ELISA Kit were used to analyze 560 samples collected in this study. It is worth noting that in this study, the ELISA value for the cell supernatant of some samples was very close to the positive judgment value, which may be due to the slow replication of some specific strains^14^. IFA using anti-P27 mAb was employed to further verify whether these samples are also ALV positive, and results showed that 13 out of 15 suspicious samples were infected, which indicated that maybe the positive judgment value of the commonly used ELISA Kit needs to be further adjusted to adapt to the characteristics of the current popular strains. However, the *env* gene of these 13 strains did not show severe gene mutation, so the molecular characteristics of them are not further analyzed in this paper, while the reason for the slow replication needs further study.

After that, a total of 71 ALV strains were identified from 560 samples with a positive rate at 12.68%, but the ALV-positive rate in different farms was significantly different from 1.67 to 29.00%, showing that there were obvious differences in the ALV infection status on chicken flocks with different genetic background and geographical location. Subsequently, all of these isolates were determined as ALV-J, while none of the other subgroups was found. This is not consistent with previous reports which showed that the popular strains in Chinese local chickens are also ALV-A, ALV-B, and ALV-K^13^, suggesting that the recent outbreak of ALV infection in Chinese local chickens was caused by the invasion of ALV-J.

Sequence comparison revealed that these 71 ALV-J isolates in this study may have a very complex genetic background. Six of them shared 100% similarity with several ALV-J strains isolated from 2009 to 2016, respectively, and these reference strains were collected from yellow broiler chicken, indigenous chicken, or commercial layers. Meanwhile, several strains have the highest similarity with reference strains isolated from the USA and Russia. These two aspects indicate that there are many sources of the ALV strains which are prevalent in the local chickens in China, while these specific flocks are likely to act as reservoirs for a variety of strains that were once in other chickens, and future research should focus on whether these strains will go out here and infect other chickens. Phylogenetic analysis further confirmed the above conclusion. The strains isolated in this study do not have strict regional or temporal characteristics like those in other chicken flocks, while some of these strains are in the same branch with reference strains of different backgrounds, showing again the diversity of the epidemic strains in the above chicken flocks. More importantly, strains isolated from Chinese indigenous chicken breeds from 2012 to 2016 are in the original evolutionary position and converge to the same evolutionary branch, while others, including ALV strains isolated in this study, are in a larger, more evolved branch. Therefore, strains that were previously prevalent in some local chicken flocks may be some ancient, long-standing strains, but now this situation has changed, and strains from different chicken flocks have entered these local chicken flocks. Meanwhile, we also found that many strains converge into novel evolutionary branches, indicating that ALV is rapidly mutating in these local chicken flocks, and it is possible to breed strains with new biological characteristics, which deserves further analysis.

On the other hand, the strains isolated in this study can also be divided into ten groups according to their genome similarity, and these different groups appeared in different flocks. Although the strains in the same group are not identical, it may be that the ancestors of these strains have experienced different evolutionary processes after entering different flocks, indicating that different chicken breeds have a great influence on the evolution speed and degree of the virus. Besides, this study also noted that there are many types of strains in the same farm, for example, there are seven groups of strains in the farm of Anyi city, revealed again that these local strains are likely to be used as reservoirs with strong enrichment capability for many different types of strains. Fortunately, the diversity of strains in a single farm did not increase morbidity and mortality, but what specific impact this will have remains to be further studied.

In conclusion, it was found that a very complex ALV-J infection broke out in several local chicken flocks in Jiangxi Province, China, which led to very serious AL symptoms and increased mortality. There were many different epidemic strains with totally different sources and backgrounds, and finally, these local flocks became reservoirs for diversified ALV-J strains. This outbreak of ALV-J is different from that of previous strains, which should be paid close attention to by relevant enterprises and parts, and effective eradication measures should be implemented as soon as possible.

## Methods

### Samples background

Jiangxi province is one of the most abundant provinces of native chicken breeds, and many excellent local breeds have been successfully developed, creating great economic profits. Since late 2018, AL-like tumor diseases suddenly appeared in those farms but few studies pay attention to it. In this study, eight different local chicken farms being all unique to Jiangxi Province, China, were selected for investigation, including Ningdu Yellow Chicken (City name + breed name), Taihe Silky Fowl, Anyi Tile-like Gray Chicken, Dongxiang Blue-shell Chicken, Chongren Chicken, Guangfeng Baier Yellow Chicken, Xiushui Black-bone Chicken and Yugan Black Chicken. As shown in Table 4, the breeding scale of eight farms investigated in this study ranged from 5000 to 10,000 chickens. The average age of suspected AL onset was around 135-day-old, and the symptoms last for 30–37 weeks with an obvious peak period about 32-week-old. The daily mortality of these farms was from 0.07 to 0.44%, and corresponding cumulative mortality was between 7.90 and 13.10%. It is worth noting that the egg production rate of these farms has decreased significantly, which is more than 20% lower than the normal level at 65%. Besides, the hatchability of breeding eggs was less affected, which was still around 80%.

**Table 4:** Production data collected from the farms investigated in this study.

Breed name	Breeding scale	Age of onset (days)	Duration (weeks)	Peak period (weeks)	Cumulative mortality (%)	Laying rate (%)	Hatchability (%)
Ningdu yellow chicken	7000	126	30	28	11.30	45.10	79.30
Taihe silky fowl	5000	141	33	30	12.20	31.10	80.60
Anyi tile-like gray chicken yiTile-likecen	10,000	130	35	29	10.40	42.40	81.50
Dongxiang blue-shell chicken	10,000	125	34	30	7.90	46.30	82.60
Chongren chicken	6000	132	36	29	8.70	48.20	80.10
Guangfeng baier yellow chicken	6000	133	35	29	8.20	50.10	81.10
Xiushui black-bone chicken	7000	143	37	31	13.10	37.30	79.30
Yugan black chicken	5000	141	36	30	12.20	33.40	78.90

According to one percent of the breeding scale, plasma samples were collected from these farms and stored for further analysis. Detailed production data, clinical signs and postmortem lesions presented by the affected chickens were recorded. For histology examination^22^, samples of liver, spleen, kidney, bone marrow and intestine from birds with suspected AL were fixed in formalin, embedded in paraffin wax and cut into sections. The sections were stained with hematoxylin and eosin and examined for lesions by light microscopy.

### ALV isolation using DF-1 cells

Virus isolation was performed in DF-1 chicken fibroblast cell line (American Type Culture Collection, Manassas, VA) maintained in our laboratory. The DF-1 cells were cultured in Dulbecco’s modified Eagle’s medium (DMEM; Invitrogen) with 12% fetal bovine serum (FBS; Invitrogen) at 37 °C in a 5% CO_2_ incubator. Lymphocytes from the plasma samples were incubated on DF-1 cells in 24 well culture plates after centrifugation at 1500×*g* for 2 min. Uninfected DF-1 cells were served as the negative control. The culture supernatant was harvested 7 days later, and the cells were passaged to the next generation. After three blind passages of infected cells, the cell supernatants and cell samples were stored at − 80 °C until analysis. After three freeze–thaw cycles, the supernatant samples from each well (described previously) were examined for the presence of ALV group-specific P27 antigen using the ALV P27 Antigen Test Kit (IDEXX; Yuanheng Laboratories) as described previously^14^.

### Indirect immunofluorescence assay (IFA)

To further identify the presence of ALV in incubated DF-1 cells, the cells with p27 results close to the judgment value of 0.2 were further detected by IFA. Briefly, cells were fixed with precooled fixation fluid (acetone/alcohol, v/v, 3/2) for 8 min and then washed in PBS, and blocked with 3% bovine serum albumin (BSA) for 1 h. After then, cells were incubated with an ALV P27-specific monoclonal antibody (mAb, provided by Qi Su) for 45 min at room temperature. The cells were then stained with fluorescein isothiocyanate conjugated goat anti-mouse antibodies (Invitrogen), according to the manufacturer’s protocol. Finally, the cells were washed, and the nuclei were stained with DAPI and mounted for confocal microscopy (Olympus FV1000).

### Genomic DNA extraction and subgroups identification

DNA was isolated from ALV-positive cells using a commercial kit (Bio-Tek, Norcross, USA), and total DNA was resuspended in 12.25 µL of DNase-, RNase-, and proteinase-free water. For subgroup verification by polymerase chain reaction (PCR), positively infected DF-1 cells were selected as a template for subgroup-specific amplification^22,32,33^ (using the primers shown in Table 5), specifically of a highly conserved region common to each ALV subgroup. Uninfected DF-1 cells were served as a negative control.

**Table 5:** Primers used in this study.

Targets	Primers (5′–3′)	Product length (bp)
ALV-J	GGATGAGGTGACTAAGAAAG	545
	CGAACCAAAGGTAACACACG	
ALV A-E	GGATGAGGTGACTAAGAAAG	295–326
	CGAACCAAAGGTAACACACG	
ALV-K	TCCAGGCCGCAACTCAC	1214
	CATACCACCACCCACGTACT	
ALV-J env	GATGAGGCGAGCCCTCTCTTTG	2300
	TGTGGTGGGAGGTAAAATGGCGT	

### env gene amplification and sequencing

The *env* gene of above-isolated strains was amplified by PCR using genomic DNA extracted from infected DF-1 cells as a template with Premix LA Taq polymerase (TaKaRa, Dalian, China) in a 50-μL reaction containing 4 μL of dNTP mixture (TaKaRa), 5 μL of 10 × PCR buffer (TaKaRa), 1 μL of Taq polymerase (TaKaRa), 2 μL of DNA solution, 1 μL of forward and reverse primers, and 36 μL of ddH_2_O. The primers and corresponding thermocycling profiles used in this study are designed in a previous study^14^ (Table 5).

The PCR products were purified by 1% agarose gel electrophoresis and then recycled by the Omega Gel Extraction Kit (USA). The purified products were then cloned into the pMD18-T vector (Transgen, China), and the resulting construct was used to transform *E. coli* DH5α cells (TaRaKa). Positive clones were sequenced by a commercial company (Shenggong, Shanghai, China), and each one was sequenced at least three times independently.

### Sequence alignment and analysis

Obtained sequences of above isolates were assembled using DNAStar (version 7.0), and multiple sequence alignment was obtained using Clustal X (BioEdit version 7.0) and Blast (NCBI). Nucleotide and deduced amino acid sequence similarity searches were performed using MEGA (version 5.0). The phylogenetic analysis was performed using the maximum likelihood method on MEGA 5.0.The sequences obtained in this study have been deposited in GenBank. A total of 172 ALV-J isolates from the past 30 years from different sources were chosen, including the ALV-J prototype HPRS-103 isolated from a white feather broiler in the United Kindom^34^, 8 isolates from the USA^35,36^, 22 isolates from white feather broilers in China^37,38^, 42 isolates from yellow feather broilers in China^19^, 51 isolates from layer hens in China^33,39–43^, 22 isolates from white feather broilers recently isolated in China^23^, and 26 isolates from indigenous chickens in China^44^.

### Ethics statement

The study protocol and all animal experiments were approved by the Animal Ethics Committee of the Institute of Animal Husbandry and Veterinary, Jiangxi Academy of Agricultural Science (2010-JXAAS-XM-01). All methods were performed following the relevant guidelines and regulations (Figure S1).

## Supplementary Information

Supplementary Information

## Data Availability

The data that support the findings of this study are available in Genbank, reference number [MT262528-MT262598].
