# Supplementary figures and images for: Diversity of Avian leukosis virus subgroup J in local chickens, Jiangxi, China
Source: Sci Rep. 2021 Feb 26;11:4797. doi: 10.1038/s41598-021-84189-7 (PMC7910287; doi:10.1038/s41598-021-84189-7)

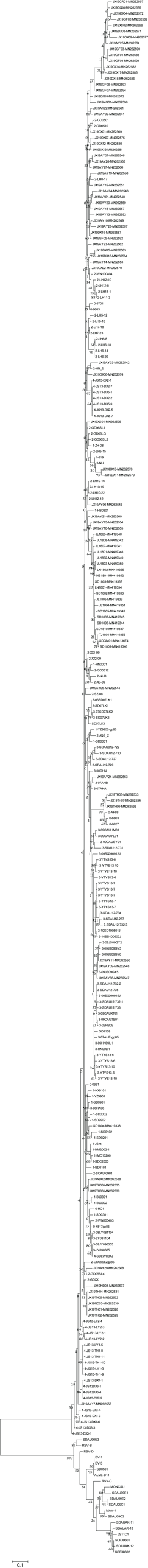

Supplement: Supplementary file 1 — Supplementary Information [file 41598_2021_84189_MOESM1_ESM.jpg]
